# Metal Oxide Nanowire Preparation and Their Integration into Chemical Sensing Devices at the SENSOR Lab in Brescia

**DOI:** 10.3390/s17051000

**Published:** 2017-05-01

**Authors:** Angela Bertuna, Guido Faglia, Matteo Ferroni, Navpreet Kaur, Hashitha M. M. Munasinghe Arachchige, Giorgio Sberveglieri, Elisabetta Comini

**Affiliations:** SENSOR Lab, Dipartimento di Ingegneria dell’Informazione, Università degli Studi di Brescia, via Valotti 9, 25133 Brescia, Italy; a.bertuna@unibs.it (A.B.); guido.faglia@unibs.it (G.F.); matteo.ferroni@unibs.it (M.F.); n.kaur001@unibs.it (N.K.); hashithamahesh@gmail.com (H.M.M.M.A.); giorgio.sberveglieri@unibs.it (G.S.)

**Keywords:** metal oxides, nanowires, chemical sensors

## Abstract

Metal oxide 1D nanowires are probably the most promising structures to develop cheap stable and selective chemical sensors. The purpose of this contribution is to review almost two-decades of research activity at the Sensor Lab Brescia on their preparation during by vapor solid (*n*-type In_2_O_3_, ZnO), vapor liquid solid (*n*-type SnO_2_ and *p*-type NiO) and thermal evaporation and oxidation (*n*-type ZnO, WO_3_ and *p*-type CuO) methods. For each material we’ve assessed the chemical sensing performance in relation to the preparation conditions and established a rank in the detection of environmental and industrial pollutants: SnO_2_ nanowires were effective in DMMP detection, ZnO nanowires in NO_2_, acetone and ethanol detection, WO_3_ for ammonia and CuO for ozone.

## 1. Introduction

The synthesis of functional materials with tailored composition, atomic arrangement and even controlled crystal shape and interfaces is one of the basic challenges in the nanotechnology research field [1]. In parallel to the pursuit of reaching the submicrometric domain with the top-down fabrication of structures, the bottom-up approach has produced over the years outstanding achievements and even today new perspectives are being regularly presented to the scientific community. As a typical case, the discovery of the allotropes of carbon, from the long-time known fullerene to the recent graphene through the elongated shapes of carbon nanotubes, have stimulated research and opened a number of potential applications [2]. Other than carbon-based materials, the production of shape-controlled nanostructures of group III–V compounds and semiconducting metal-oxides such as ZnO, SnO_2_, In_2_O_3_ was investigated and it stimulated during the last two decades a revival of interest [3], finding many applications and implementations in energy-harvesting and conversion, sensing and light-generating devices [4]. Nano-sized crystals of semiconducting oxides, particularly in the form of elongated crystals (nanowires or one-dimensional nanostructures) are indeed promising for the fabrication of miniaturized functional devices, where the single nanocrystal could act as the active part of the system. For these systems, the space-charge concentration for both ions and electron is so greatly affected by interfaces that the electrical/thermal conductivity radically changes. The resulting unusual properties may be exploited under the requirement that the nanomaterials preserve an acceptable degree of stability [5,6].

This scenario could be realized provided that the fabrication methods fulfill the requirements of reproducibility, integration with the established industrial processes and economical sustainability. Thus, the vapor phase methods presented and used for nanowire fabrication are among the most effective in terms of scalability and integration, and they are highly considered [7,8]. 

This paper discusses specifically the methods and scientific results achieved by the SENSOR Lab at Brescia in the preparation of metal-oxide nanowires for gas sensing applications.

## 2. Nanostructures Preparation Methods 

For the preparation of 1D nanostructures, the growth process must occur at a higher growth rate along a preferable crystallographic direction. In order to grow one-dimensional metal oxide nanostructures, bottom-up techniques have been most widely employed. Commonly used growth techniques are hydrothermal growth [9], sol-gel method [10], template assist growth [11], evaporation-condensation growth [12], etc. Among these, evaporation-condensation and thermal oxidation have been extensively used to grow 1D nanostructures such as nanowires. They are easy, simple and cheap methods to obtain single crystal nanostructures [13,14]. We will report in the following subsections the research achievements of the SENSOR laboratory in these two preparation techniques. 

### 2.1. Evaporation-Condensation Process

The evaporation-condensation growth process can be described through to two different mechanisms: vapor-solid (VS) or vapor-liquid-solid (VLS). VS growth involves two material phases: vapor and solid. In this process the crystallization of nanowires starts with a direct condensation from the vapor phase without using any catalyst. In this mechanism, source materials are vaporized under high temperature and low-pressure conditions and directly condensed on the target substrates placed in a low temperature region. On the other hand, the VLS mechanism is named after the three different material phases involved in the growth process, i.e., vapor, liquid and solid. This growth mechanism was already described in 1964 by Wagner [15]. The liquid phase refers to the liquid metal catalyst, which acts as a preferable site for the adsorption of source material vapors. Usually these catalysts are noble metals like palladium, platinum, gold or ruthenium. These catalysts play a very important role for the growth of 1D nanostructures as it’s possible to determine the nanowires’ lateral size from the catalyst cluster dimensions. Furthermore, as the vapors of source material are continuously provided, the super-saturation state can be reached and the material starts to condense as 1D crystalline nanostructures. 

**Figure 1:**
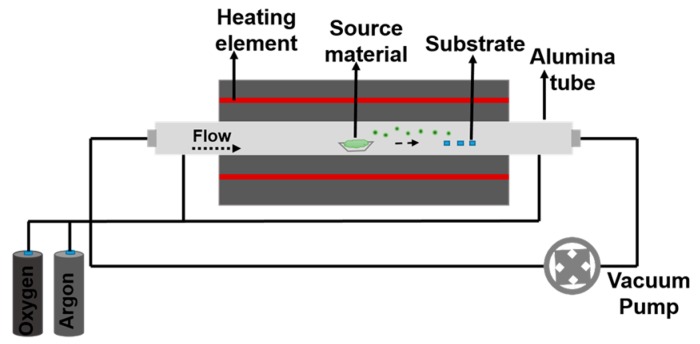
Schematic diagram of the growth experimental setup (Tubular Furnace).

This technique was used at SENSOR laboratory for the fabrication of different metal oxide nanostructures, mainly nanowires, using custom made tubular furnaces. The typical setup of an evaporation-condensation process consists of a vacuum sealed furnace in which the source material is placed in the middle of an alumina tube and heated at a certain temperature to induce its evaporation. These tubular furnaces can reach a maximum temperature of 1500 °C. The vapors of the source material are transported with the help of carrier gases (oxygen and argon) that condense on the substrate placed at lower temperatures as compared to the evaporation one. The substrate can be bare or covered with the metal catalyst depending on which mechanism is used. The catalyst is deposited on a substrate using magnetron sputtering. While the furnace reaches the evaporation temperature, the flow is kept in reverse direction (from substrates to powder) in order to avoid any undesired condensations that could be not at the right deposition conditions. When the furnace reaches the desired temperature, the flow is reversed in the direct direction (from powder to the substrates) and the deposition starts. The deposition time is also a very important parameter and it can be changed according to the desired amount of nanostructures on the substrate. Figure 1 shows the schematic of a tubular furnace setup used for depositions at SENSOR.

As mentioned, VLS growth was carried out with different catalysts such as gold, platinum, palladium, ruthenium and different substrates (alumina or silicon). It is important to point out that the growth of nanowires depends on many parameters such as condensation temperature, substrate temperature (St), pressure (P), gas flow carrier and time of deposition. Therefore, different growth conditions have been used for each material.

### 2.2. Thermal Oxidation Process

Thermal oxidation is another technique that can be used to prepare 1D metal oxide nanostructures. This technique has many advantages compared to other ones. It is a simple technique with relatively low equipment costs because it just requires a tubular furnace and a primary vacuum pump for the eventual need of working at pressures lower than the atmospheric one. It guarantees high product yields with no risk of cross contamination, due to the low working temperatures involved in the process [16]. Moreover, this technique is scalable for mass production and it is highly reproducible because all the steps can be automated and are independent from the operator’s contribution and skills [14]. 

**Figure 2:**
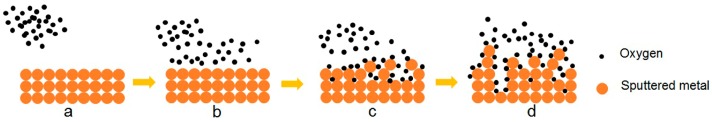
The growth mechanism of thermal oxidation. (**a**) Presence of oxygen ions in the atmosphere; (**b**) Surface reactions between oxygen ions and metallic atoms; (**c**) Diffusion of oxygen into the metal and nucleation of nanowires; (**d**) Growth of nanowires and oxidation.

Oxide whisker growth with thermal oxidation is a well-known growth mechanism [17]. Figure 2 shows the different steps required in this technique to prepare metal oxide nanostructures. Surface reactions are occurring with oxygen atoms present in the atmosphere due to high thermal energy (Figure 2a,b). Mechanical stress promotes the nucleation due to oxygen atom diffusion inside the metal layer [16]. In-phase tensile stresses can effectively promote NW growth by significantly increasing the NW density (Figure 2c) and furthermore, the topmost layer is highly porous, defective and oxidized [18,19]. Metal atoms find two pathways (lattice and gran-boundary diffusion) to reach the oxide/air interface for further oxidation. Lattice diffusion results increase the oxide layer thickness, while grain-boundary diffusion results in the formation of NWs (Figure 2d) [20,21]. Furthermore, the morphology of the nanostructures strongly depends on different parameters such as pressure, temperature, atmosphere composition, oxidation time and type of material. The growth process consists in two phases: metal deposition on the substrate, which can be performed by magnetron sputtering, thermal evaporation or electro-deposition, and thermal oxidation in a tubular furnace.

We summarize the achievements of the growth of different metal oxides nanowires (either *n*- and *p*-type) such as In_2_O_3_, SnO_2_, ZnO, CuO, WO_3_ and NiO that were successfully grown using evaporation-condensation and thermal oxidation techniques at the SENSOR Laboratory. The nanostructures achieved have been used in many applications such as gas sensors, optical sensing and solar cells.

## 3. Conductometric Gas Sensors

**Figure 3:**
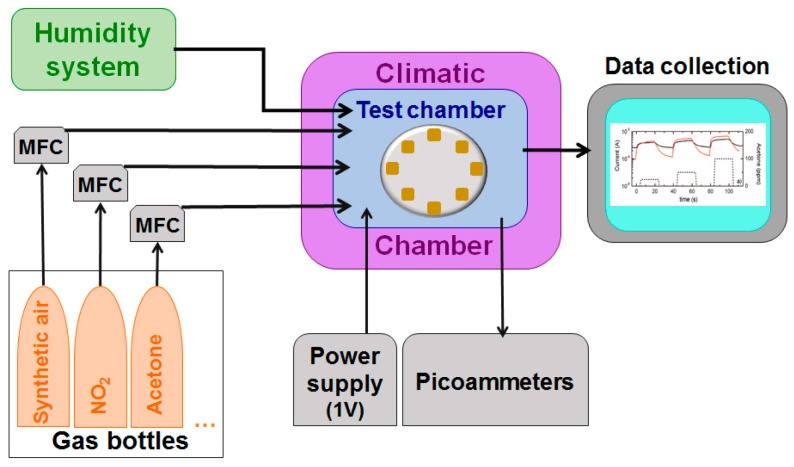
Schematic diagram of gas sensing setup.

Gas sensing tests were performed on chemical sensors in a homemade stainless-steel chamber, which is kept at constant temperature in a climatic chamber. The conductometric response of the devices was investigated by a flow-through technique with controlled relative humidity (usually 40%). Experiments were carried out in an atmosphere composed of traces of gases, coming from certified bottles, dispersed in dry synthetic air and mixed with mass flow controllers (MFC) to obtain the desired atmosphere composition. Usually, the flow is maintained constant at 0.3 L/min during the measurement and the devices are biased at 1 V. An extensive setup description is reported in [22]. Figure 3 shows a schematic diagram of gas sensing setup.

The sensor response is defined as a variation of the electrical resistance, or conductance, which occurs when the device is exposed to a gas. This variation is calculated in two different ways, depending on whether the material is an *n*-type or a *p*-type semiconductor. Sensor response is calculated using the following relations, in case of oxidizing (1) and reducing (2) gases, respectively, for an *n*-type semiconductor:

(1) $$Response=\frac{R_{gas}-R_{air}}{R_{air}}=\frac{G_{air}-G_{gas}}{G_{gas}}$$

(2) $$Response=\frac{G_{gas}-G_{air}}{G_{air}}$$

where $G_{air}$ is the baseline conductance of the sensor exposed to synthetic air, and $G_{gas}$ in presence of gas. These relations were preferred since in presence of an oxidizing gas the device conductance decreases from the baseline value up to a new equilibrium state for an *n*-type semiconductor. The opposite occurs when *n*-type conductometric sensors are exposed to a reducing gas. 

In the case of *p*-type semiconductors, instead, the response may be calculated as:

(3) $$Response=\frac{R_{air}-R_{gas}}{R_{gas}}=\frac{G_{gas}-G_{air}}{G_{air}}$$

(4) $$Response=\frac{G_{air}-G_{gas}}{G_{gas}}$$

Since the conductance increases in the presence of oxidizing gas (3) and it decreases with a reducing gas (4).

Ionosorption of gaseous species that occurs on the metal oxide surface is directly connected to the sensing mechanism. Oxygen and water play an important role since they are the principal ionosorbed species under ambient air conditions. The first one may ionosorb over the sensor surface in two different forms: molecular ($O_{2}^{-}$) and atomic ($O^{-}$) oxygen. This happens when the temperature is in the range 100 to 500 °C. In particular $O_{2}^{-}$ predominates up to 200 °C, because of its lower activation energy, while at higher temperatures the $O^{-}$ form dominates [23,24,25]. In case of a reducing gas, the interaction with the surface may lead to its oxidation due to the reaction with the ionosorbed oxygen. For example, in the case of CO, the reaction results in the formation of CO_2_ combined with the release of electrons by the surface states to the conduction band [25,26,27]. This reaction is explained by the following reactions [23]:

(5) $${\mathrm{CO}}_{\mathrm{gas}}\to {\mathrm{CO}}_{\mathrm{ads}}$$

(6) $${\mathrm{CO}}_{\mathrm{ads}}+O_{\mathrm{ads}}^{-}\to {\mathrm{CO}}_{2,\mathrm{gas}}+e^{-}$$

The change in the value of the metal oxide electrical conductance is due to the consumption of ionosorbed oxygen. More in detail, the change in the ionosorbed oxygen density leads to an increase of the device electrical conductance. In the case of oxidizing gases (like NO_2_), which causes a decrease of the electrical conductance of metal oxides, the reactions may be explained by the following relations [23,26]:

(7) $${\mathrm{NO}}_{2,\mathrm{gas}}⇄{\mathrm{NO}}_{2,\mathrm{ads}}$$

(8) $$e^{-}+{\mathrm{NO}}_{2,\mathrm{ads}}⇄{\mathrm{NO}}_{2,\mathrm{ads}}^{-}$$

The conductance of the sensor is reduced while the surface potential increases because of the occupation of the surface states, which are much deeper in the band gap of oxygen. Concerning water molecules, their chemisorption can form a “hydroxylated surface”, where the metal cation is linked to OH^−^ ion and the oxide anion to H^+^ ion. The presence of water vapor produces an increase in surface conductance [26]. 

## 4. Surface Morphology Investigation of Nanowires

Scanning electron microscopy (SEM) is the primary tool to investigate the morphology of nanowires. The SENSOR Laboratory has published number of reports over the years on the fabrication of different *n*-type and *p*-type metal oxides nanowires using evaporation-condensation techniques for gas sensing. The following are some interesting reports that were published over the years on different nanostructure metal oxides, in particular for gas sensing.

### 4.1. Evaporation-Condensation Process

#### 4.1.1. Indium Oxide

**Figure 4:**
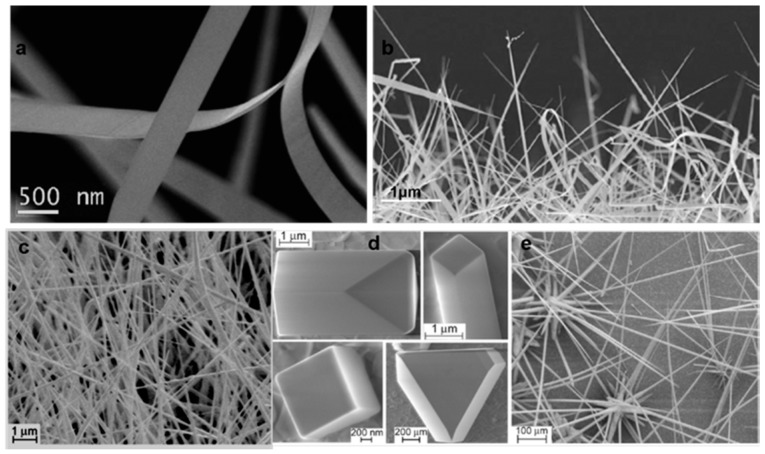
(**a**) SEM images of nanosized cubes and nanowires of indium oxide; (**b**) SEM images of nanosized cubes and nanowires of indium oxide; Reprinted from [29] with permission form Elsevier, copyright (2006); (**c**) SEM image at (S_t_ = 750 °C) thin and densely packed nanowires obtained at (S_t_ = 750 °C); (**d**) SEM image at (S_t_ = 900 °C) growths at (S_t_ = 900 °C) in the form of form of microcubes and regular polyhedrons; (**e**) Images at (S_t_ = 1000 °C) shows formation at (S_t_ = 1000 °C) of thick and dispersed microwires. Reprinted from [28] with permission from Elsevier, copyright (2007).

Indium oxide (In_2_O_3_) nanowires were grown using both the VLS and VS techniques [28,29]. The deposition was performed at a very high evaporation temperature (E_t_), i.e., at 1500 °C. Different types of 1D nanostructures such as nanobelts and nanowires were obtained depending on the condensation temperature or on the type of catalyst present. For the VLS process a Pd catalyst was used, while in VS growth, indium (In) itself was used for seeding on alumina substrates. In the very first report, In_2_O_3_ nanowires were grown on an alumina substrate at E_t_ = 1500 °C, P = 200 mbar and S_t_ = 1100 to 800 °C [29]. Figure 4a,b show SEM images of In_2_O_3_ nanostructures grown on bare alumina substrates using the VS technique. Clearly, different crystalline structures were obtained, ranging from coarse and equiaxial grains to whisker-like structures, nanowires and nanobelts. These different nanostructures show the influence of the substrate temperature on the shape of In_2_O_3_ crystalline structures. The diameter of the nanowires was less than 100 nm. Furthermore, Vomiero et al. [28] presented a report on growth of In_2_O_3_ nanowires with the same technique, but using In particles as seeds and a Pd catalyst-deposited alumina substrate instead of a bare one.

**Figure 5:**
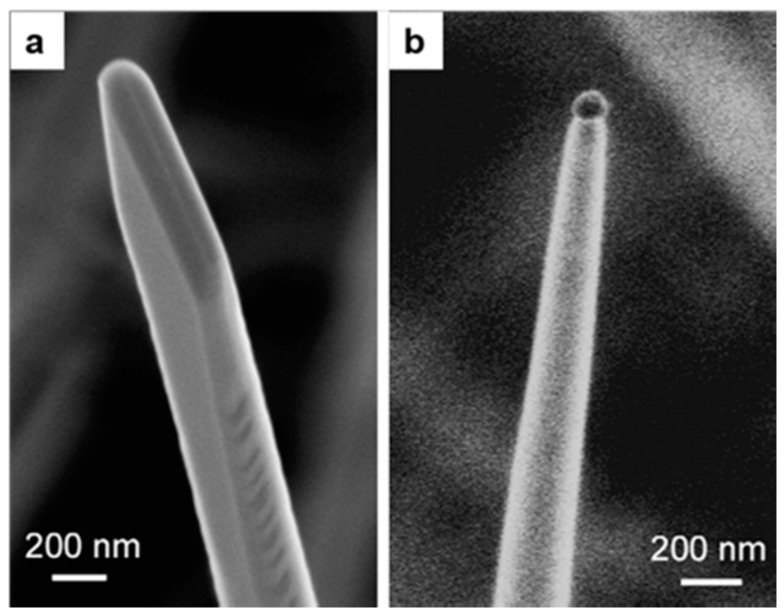
SEM images of the terminations of two nanowires synthesized by templating the substrate with either In (**a**) or Pd (**b**). Reprinted from [28] with permission from Elsevier, copyright (2007).

The E_t_ temperature was also in this case 1500 °C, while the substrate temperature S_t_ ranged from 700 to 1000 °C. Figure 4c–e show SEM images of different In_2_O_3_ nanostructures made using In particles as seeds and grown at different S_t_. The formation of microwires was observed at S_t_ = 1000 °C (region e). On the other hand, in the lower temperature region d (S_t_ = 900 °C) the formation of microcubes, parallelepipeds and regular polyhedrons was observed, while in region c (S_t_ = 750 °C) thin and densely packed nanowires with lengths up to tens of microns were grown. In this report the comparison on In_2_O_3_ nanowires growth using In as seeding material and Pd as catalyst was presented. Figure 5a,b shows SEM images of the terminations of two nanowires synthesized by templating the substrate with In (Figure 5a) and Pd (Figure 5b). The nanowire grown with the In seeding has a sharp lateral facets and pyramidal termination (Figure 5a). The average lateral dimension of the nanowires was found to be 110 ± 20 nm. A pyramidal tip appeared on top of the nanowire, indicating that the nanowire growth was driven by a VS growth mechanism, without any external catalytic mechanism [30,31,32]. 

**Figure 6:**
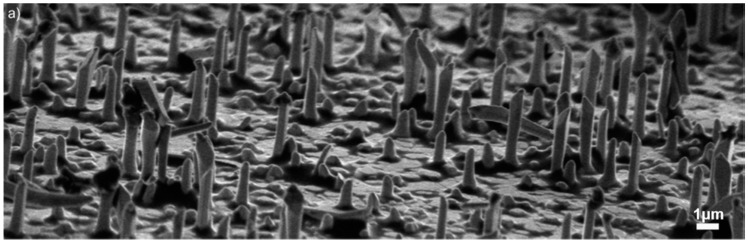
Prospect view of typical In_2_O_3_ nanowires showing the preferential growth direction, normal to the sapphire substrate. Reprinted from [35], with permission from American Chemical Society, copyright (2010).

Pd seeding results in the growth of nanowires terminated by a metallic droplet, and with microfaceted lateral sides (Figure 5b). The thinnest nanowires are as narrow as 20 nm. However, the distribution of lateral dimensions for nanowires was found to be on average 210 nm. The presence of the catalytic tip clearly indicates a dominating VLS mechanism in this case [15,33]. Another interesting report was published the same year on the detailed controlled growth study of In_2_O_3_ nanowires grown with the VS method using In seeding [34]. Further, in 2009 Vomiero et al. [35] reported an insight into the formation mechanism of one-dimensional indium oxide wires grown with VLS assisted by a gold catalyst. The report described quantitative investigations on the nucleation and growth of indium oxide nanowires on single crystal substrates, mediated by catalytically active gold particles. Figure 6 shows an overview SEM image of In_2_O_3_ nanowires after 10 min condensation at different temperatures (Ar = 75 sccm, P = 100 mbar). Each nanowire has a rectangular cross-section and gold nanoparticles were found on the tip of the nanowires. There was no significant change observed in the size of the gold clusters below 1235 K. 

#### 4.1.2. Tin Oxide

**Figure 7:**
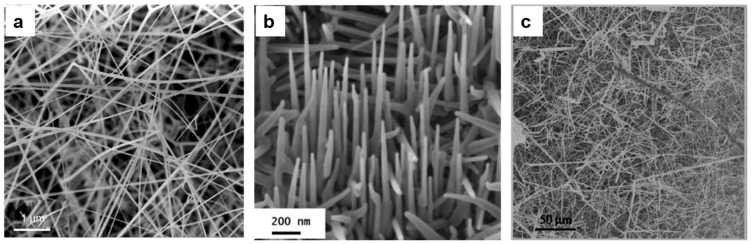
(**a**) VS growth of SnO_2_ nanowires over polycrystalline alumina. A very high aspect ratio for the nanostructures was obtained; (**b**) VLS growth of SnO_2_ nanowires over polycrystalline alumina assisted by dispersion of Pt nanoparticles. Growth of aligned nanowires. Reprinted from [36] with permission of Springer; (**c**) General SEM view of the synthesized SnO_2_ nanowires. Reprinted from [40] with permission from Elsevier, copyright (2008).

SnO_2_ is a one of the most investigated materials in the field of gas sensors in the form of different nanostructures. SnO_2_ nanowires were grown on different substrates such as alumina, silicon and SiC. Pt is the most commonly used catalyst in the literature for the growth of SnO_2_ nanowires. In 2007 Comini et al. [36] reported a detailed study for the fabrication of SnO_2_ nanowires on bare alumina substrates using Pt catalysts. SnO_2_ powder was used as source material and heated at an evaporation temperature of 1370 °C while the pressure was kept at 100 mbar inside the alumina tube. Figure 7a,b show the SEM images of SnO_2_ nanowires grown on bare and Pt catalyst- covered alumina substrates. The nanowires in Figure 7a shows very high aspect ratios (the ratio between the length and width of the nanowire), i.e., more than 500. No epitaxial relationship between the orientation of the wire and the alumina grains has been observed, while the presence of Pt nanoparticles affects the growth of the nanowires. Moreover, the S_t_ (around 470 °C) was found to promote nucleation on catalyst-deposited substrates, while a temperature of 330 °C was required for catalyst-free growth. The higher temperature allowed the formation of melted Pt-Sn clusters, which promoted the nucleation of the nanowires, therefore, the growth of the wire was of VLS type. In 2008, a number of papers were published on the growth of SnO_2_ nanowires using the same method for conductometric and optical gas sensing applications [37,38,39]. The same E_t_ = 1370 °C was used and the deposition was carried on silicon and SiC substrates, while the substrate temperature ranged between 400 and 500 °C. 

A report was published on the growth of SnO_2_ nanowires (Figure 7c) using an evaporation-condensation technique, but tin monoxide powder was used as source material instead of tin dioxide [40]. In the deposition process tin monoxide was placed at the center of the quartz tube and the alumina substrates were positioned in the lower temperature region, then the system was pumped down and the temperature raised to 300 °C under vacuum. A subsequent temperature ramp to 900 °C was imparted keeping a 100 sccm flow of Ar/H_2_ at 300 mbar in order to prevent the oxidation of tin monoxide. At temperatures higher than 750 °C a complete dissociation of tin monoxide into tin and tin dioxide took place. The carrier gas transports tin vapors and due to the lower temperature of the substrates they condensate in liquid droplets with dimensions ranging from tens of nanometers to microns. The temperature is then slowly decreased to 870 °C and an Ar/O_2_ flow introduced. Oxygen reacts with tin droplets and forms SnO_2_ nuclei that then develop nanostructures. The nanowires found are very irregular and their widths range from 20 to 250 nm while their lengths are up to 200 μm.

**Figure 8:**
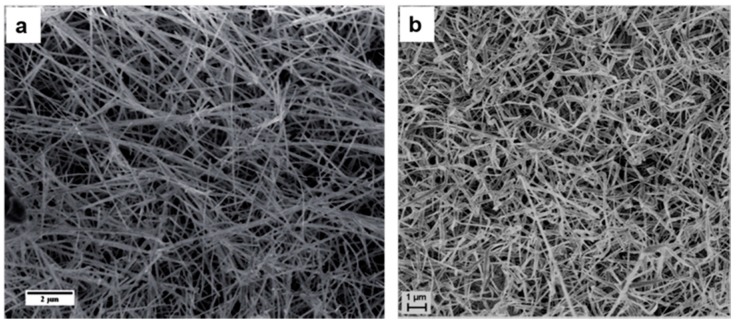
SEM images of SnO_2_ nanowires. (**a**) Using Au catalyst; Reprinted from [43] with permission from Wiley, copyright (2014); (**b**) Using Ag catalyst. Reprinted from [44].

Additional reports were also published from 2009 to 2012 about the growth of SnO_2_ nanowires using evaporation-condensation techniques and the same growth conditions for chemical sensing and e-nose applications [41,42]. In 2014 two reports were published on the growth of SnO_2_ nanowires using evaporation condensation with SnO_2_ powder as source material but a different catalyst was used to grow the nanowires. In the first report, the authors showed the results of SnO_2_ nanowires grown on SiC substrate using Au as a catalyst [43]. Deposition parameters (E_t_ = 1370 °C, P = 100 mbar, S_t_ = 500 °C) were similar to those first reported by Comini et al. [36]. Figure 8a shows a SEM image of the SnO_2_ nanowires. The measured widths of the nanowires were found to lie between 100 nm and 1 μm while their lengths reached up to 20 μm. 

In the second report, the growth of SnO_2_ nanowires was performed using Ag as a catalyst and the substrates were kept at S_t_ = 850 °C [44], keeping the other growth parameters as in [43]. The morphology of the resulting SnO_2_ nanowires is shown in Figure 8b. The results show that nanowires were irregular in shape and very thick. It was also found that the ratio between their length (several microns) and width (less than 100 nm) was very high.

#### 4.1.3. Zinc Oxide

**Figure 9:**
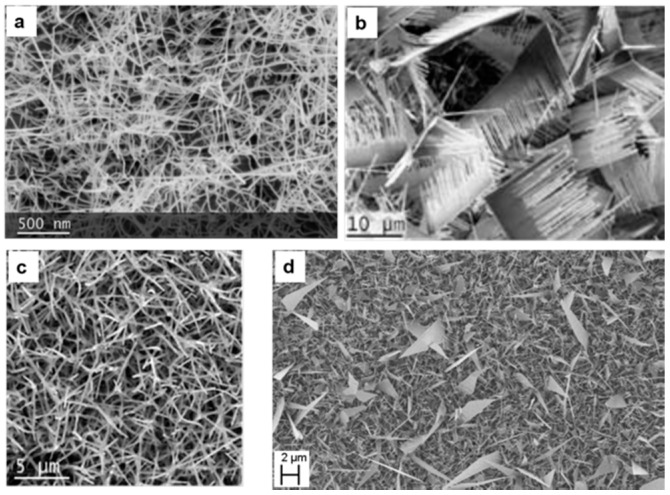
SEM images of (**a**) the ZnO nanowires over the alumina substrate; Reprinted from [45] with permission from IOP Publishing, all rights reserved; (**b**) the dispersion of ZnO nanocombs over the substrate; (**c**) the ZnO nanowires produced using Au catalyst. Reprinted from [46] with permission of Springer; (**d**) ZnO nanowires using Au catalyst at different S_t_. Reprinted from [47] with permission from IOP Publishing, all rights reserved.

In the field of nanostructure-based gas sensors, ZnO is one of the most studied materials [23]. Concerning zinc oxide nanostructures grown with the evaporation condensation technique, the condensation temperature for obtaining nanowires is relatively low compared to other metal oxides. In 2007 Comini et al. [45] reported on ZnO nanowires grown on alumina substrates without any catalyst as shown in Figure 9a. ZnO powder was heated at E_t_ = 1370 °C while P = 100 mbar and Ar = 75 sccm (carrier gas flow) was maintained. The S_t_ temperature was found to lie between 400 and 500 °C. The nanowires showed a dense arrangement which covered the substrate and appeared uniform in morphology. The length of the nanowires spanned several micrometers, while preserving a uniform lateral size on the order of tenths of a nanometer. 

The same year Comini et al. [46] also published another interesting report on the growth of ZnO nanowires using Zn powder as source material instead of ZnO. The deposition of nanowires was performed in a tubular furnace, but the E_t_ temperature was lower as compared to the one used to evaporate ZnO powder. The Zn powder was heated up to 600 °C at ambient pressure with 20% oxygen and a carrier Ar flow of 500 sccm. The alumina substrates were placed at a distance ranging from 3 to 5 mm from the source material. The results from this deposition showed the growth of nanocombs (see Figure 9b). Nanocombs were grown over a large area of the substrate. The SEM images also confirmed the high yield achieved through the evaporation method as the substrate was covered by numerous nanocombs. TEM analysis revealed the regular and parallel arrangement of the nanowires. The width of the nanowires averaged 176 ± 15 nm. Variation of the deposition parameters and also of the source-substrate geometry resulted in the formation of different types of nanowires. 

Figure 9c shows that the production of micrometric-sized nanowires can be achieved in the case of high deposition rates. Furthermore reducing the condensation rate through an increase in the source-substrate distance, results in a dispersion of very thin wires. Moreover, in 2009 other reports were published on the growth of ZnO nanowires with the VLS method using Au as catalyst [47,48]. However, the growth parameters were similar to the ones reported in [45]. In one of the reports the authors showed the growth of film consisting of ZnO nanowires (Figure 9d) [47]. The S_t_ temperature (400 to 500 °C) range for this growth was a little higher than the one in the other report [48]. Figure 9d shows that the top surface of the layers exhibits larger structures (with an average size on the order of 1 μm) covering a dense entanglement of smaller wire-like ZnO structures whose diameters are on the order of a few tenths of nanometers. 

**Figure 10:**
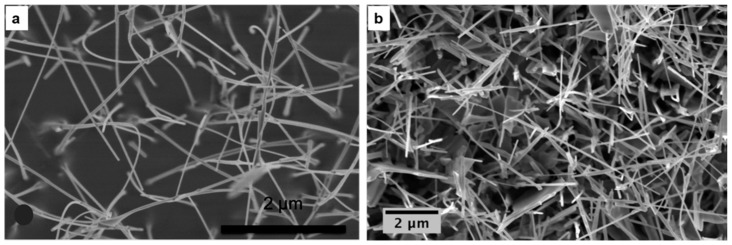
SEM images of ZnO nanowires. (**a**) Using Pt catalyst; Reproduced from [49]; (**b**) using Au catalyst. Reproduced from [51] with permission from the Royal Society of Chemistry.

More reports were published from 2011 to 2013 on ZnO nanowire growth by evaporation-condensation [49,50,51] for many different applications such as gas sensing and UV photoluminescence, etc. In one of the reports, the authors showed the growth of ZnO nanowires as shown in Figure 10a using Pt as catalyst [49]. The S_t_ (660 °C) was found to be higher as compared to the one used with Au catalyst. TEM analysis shows that the nanowires are single crystalline ZnO with a wurtzite structure. Figure 10b shows the ZnO nanowires grown on sapphire substrates catalyzed with gold nanoparticles at a lower condensation temperature S_t_ = 350 °C [51]. The nanowires have a random orientation with diameters ranging from 50 to 100 nm and are several microns in length. In addition, planar structures like platelets were also observed on the substrate. Nanowires were found to be single crystalline in nature. From all these reports it has been observed that the growth of ZnO nanowires is very sensitive to the substrate temperature and to the type of substrate.

#### 4.1.4. Nickel Oxide

**Figure 11:**
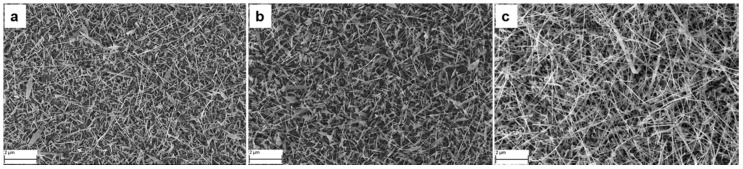
SEM images of NiO nanowires. (**a**) Using Pt catalyst; (**b**) Using Pd catalyst; (**c**) Using Au catalyst.

Many reports have been published in the literature on *n*-type metal oxide nanowires for chemical sensing applications [23,36], but there are only a few data available on *p*-type metal oxide nanowires for chemical sensing. Recently, in 2016, a report was published for the first time by the SENSOR laboratory on the growth of NiO nanowires using evaporation-condensation method. In this report Kaur et al. [52] presented the detailed optimization of the growth of NiO nanowires on alumina substrates using different catalysts (Pd, Pt and Au). Moreover, the effect of different St using different flows of Ar was also studied. NiO powder was used as source material and it was heated at E_t_ = 1400 °C. Figure 11a–c show a comparison between NiO nanowires grown using Pt, Pd and gold catalyst, respectively. The best nanowire growth was found when Au catalyst and E_t_ = 1400 °C, Ar = 100 sccm, P = 1 mbar and at S_t_ = 930 °C were used as growth conditions. The deposited nanowires were thin, long and had a denser morphology with diameters ranging from 16 nm to 50 nm. Further characterization revealed that the NiO nanowires have a bunsenite crystalline structure.

### 4.2. Thermal Oxidation Process

WO_3_, CuO and ZnO nanostructures have been prepared at SENSOR by thermal oxidation starting from metallic layers sputtered on alumina substrates. RF magnetron sputtering was preferred to deposit thin films of metallic tungsten, zinc and copper layers thanks to its compatibility with the IC industry and its reliability. Table 1 shows the deposition conditions of metallic thin films. The gas flow was kept constant (7SCCM) for all depositions, resulting in a pressure inside the chamber of 5.3 × 10^−3^ mbar.

In order to carry out the oxidation process, the substrates covered by W, Cu and Zn films were placed in a PC-controlled tubular furnace. The morphology of the nanostructure is strongly dependent on the oxidation temperature, the oxygen/argon ratio of the introduced atmosphere as well as the working pressure. These parameters were varied to investigate their influence on morphology. Table 2 shows the optimal parameters for the preparation of each material by thermal oxidation. The oxidation temperature has a strong influence on NW growth and it is an important parameter that can be used to control the sample morphology. Higher oxidation temperatures were used to achieve ZnO nanostructures and lower ones for CuO. The growth for both materials was obtained at atmospheric pressure, while lower pressures and high temperatures are necessary for the formation of WO_3_ NWs. 

#### 4.2.1. Copper Oxide

CuO, which features an interestingly narrow band gap of 1.2 eV [53,54,55] has been used in chemical sensing applications, as a component in high-T_c_ superconducting materials [56] and CuO is also an antiferromagnetic material [57]. CuO NWs has been synthesized through several methods. Among these, thermal oxidation may be a direct and clean approach to grow CuO NWs [58]. Different research groups have synthesized CuO NWs by oxidizing Cu foils under different annealing temperatures and atmospheric compositions [59,60]. 

In 2013 Zappa et al. [61] reported the preparation of copper oxide nanowires directly on sensing transducers by thermal oxidation. A metallic copper layer was deposited on alumina substrates by RF magnetron sputtering at RT, 200 °C, 300 °C and 400 °C. The layer thickness was changed from 300 nm to 3 µm and all other parameters were kept constant (7SCCM argon flow, 50 W argon plasma and pressure of 5.5 × 10^−3^ mbar). 

Copper is a very reactive metal and in an ambient atmosphere it reacts with oxygen producing a thin layer of copper oxide. This thin layer of copper oxide is detrimental for NW growth, therefore its removal before the thermal oxidation step is essential. Wet chemical etching [62] and plasma etching were used [63]. The latter approach is less aggressive and an etching with argon at 15 W for 5 min efficiently removes the native surface oxide. 

In the specific case of copper oxide, the oxidation mainly consists of two steps: oxidation of metallic copper to Cu_2_O and oxidation of Cu_2_O film to CuO forming NWs. The compressive stress produced at the CuO/Cu_2_O interface, followed by a surface diffusion of cations on the sidewall of the nanowires, has been proposed as a possible explanation for CuO nanowire growth [59]:

(9) $$\mathrm{Cu}+O_{2\;}\to {\mathrm{Cu}}_{2}O$$

(10) $${\mathrm{Cu}}_{2}O+O_{2}\to \mathrm{CuO}$$

**Figure 12:**
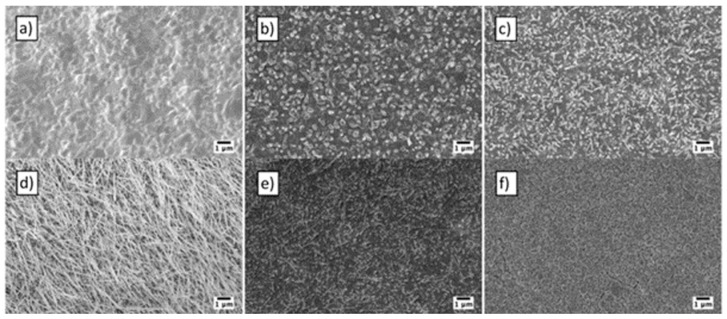
Influence of oxidation temperature: (**a**) 600 °C; (**b**) 500 °C; (**c**) 400 °C; (**d**) 300 °C; (**e**) 250 °C; and (**f**) 200 °C. The atmosphere composition was 80% O_2_ and 20% Ar, while the sputtering time was 3 h at RT (1.8 μm). Reprinted from [61] with permission from Elsevier, copyright (2013).

**Figure 13:**
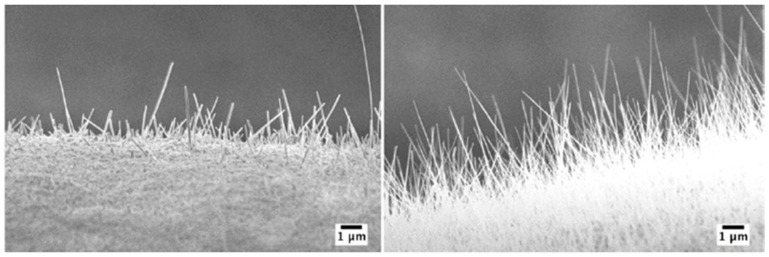
NWs comparison between (**left**) 400 °C and (**right**) 300 °C. The atmosphere composition was 80% O_2_ and 20% Ar. Substrates were Cu sputtered on alumina at RT for 5 h. Reprinted from [61] with permission from Elsevier, copyright (2013).

The substrate temperature during metallic Cu layer deposition, the oxidation temperature and atmosphere composition have a strong influence on nanowire growth. Figure 12 shows the variation of morphology when the oxidation temperature changes from 200 °C to 600 °C in the tubular furnace. The optimal oxidation temperature for growth of NWs ranged between 250 °C and 500 °C. Figure 13 shows that vertically aligned NWs were grown at 400 °C and randomly arranged NWs were present at 300 °C. 

Figure 13 shows the oxidation of two different samples at 400 °C and 300 °C with 80% O_2_ and 20% Ar. The length and diameter of the CuO NWs are mainly dependent on the oxidation temperature. Oxidation at 300 °C shows a very high aspect ratio (ratio between the length and width of the nanowire) compared to oxidation at 400 °C. 

#### 4.2.2. Zinc Oxide

**Figure 14:**
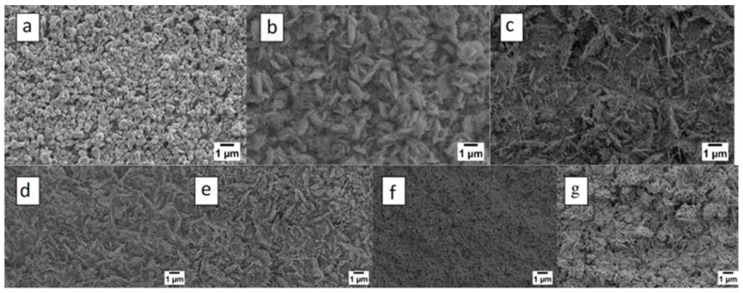
Influence of oxidation temperature: 200 °C (**a**), 300 °C (**b**), 400 °C (**c**), 500 °C (**d**), 600 °C (**e**), 700 °C (**f**) and 800 °C (**g**). The atmosphere composition was 100% O_2_, while the metallic zinc sputtering time was 3 h (4.5 μm) at RT. Reprinted from Ref. [16] with permission from IOP Publishing. All rights reserved.

In 2013 Zappa et al. [16] reported ZnO nanostructures growth by thermal oxidation directly on an alumina substrate. Metallic Zn films were deposited using a RF magnetron (7SSCM argon flow, 50 W argon plasma and pressure of 5.3 × 10^−3^ mbar). Zinc oxide nanostructures obtained by thermal evaporation are less defined (in comparison with the ones prepared by VLS), as can be seen in Figure 14. The formation process depends on the oxidation temperature and oxidation time. ZnO NWs grow between 400 and 600 °C. Relatively large particles are obtained below 400 °C and above 600 °C. At 200 °C the morphology of the resulting nanoparticles with higher dimensions compared to the nanoparticles obtained at 700 °C and 800 °C changes completely and a porous-like structure is obtained (Figure 14g). 

**Figure 15:**
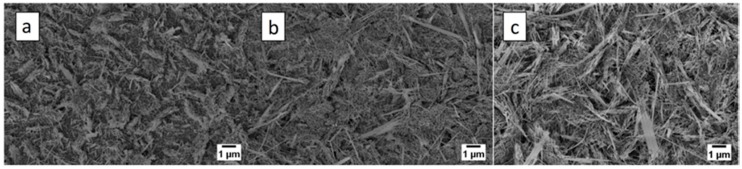
Influence of oxidizing atmosphere at 400 °C: (**a**) 100% O_2_; (**b**) 80% O_2_-20% Ar; (**c**) 50% O_2_-50% Ar. Reprinted from [16] with permission from IOP Publishing. All rights reserved.

Another important parameter that affects the morphology is the atmosphere composition. Figure 15 shows the influence of oxygen at 400 °C when the oxygen ratio in the atmospheric composition is changed. Decreasing the oxygen ratio in the atmospheric composition promotes the density and the length of the nanowires [16]. Although this parameter has effects on morphology at 400 °C, there is no macroscopic effect on morphology at 300 °C and below.

#### 4.2.3. Tungsten Oxide

WO_3_ is *n*-type semiconductor that exhibits a wide band gap of 2.7 eV [64]. It is widely studied because of its many interesting physical and chemical properties; in particular WO_3_ nanostructures have been extensively studied for gas sensing applications due to their fast response, high sensitivity and high thermal stability [65,66]. Because of these properties many efforts were made to synthesize WO_3_ in the form of nanoparticles, one-dimensional nanorods, two-dimensional nanoplates and other shapes [67,68,69,70]. Most of the syntheses rely on hydrothermal [71] and sol-gel [72] methods, while there isn’t a great amount of data for thermal oxidation.

In 2015 Zappa et al. [22] published a study on the growth of WO_3_ NWs on alumina substrates. The effect of film thickness and deposition temperature on the morphology have been studied. The metallic tungsten layer thickness was 18 nm and 180 nm and the film deposition temperature 200 °C and 300 °C, while the plasma conditions were kept constant (100 W argon flow and 5.5 × 10^−3^ mbar of pressure). The oxidation step was performed in a tubular furnace at 550 °C for one hour and at a pressure of 0.8 mbar with an oxygen flow of 2SCCM inside the tubular furnace.

**Figure 16:**
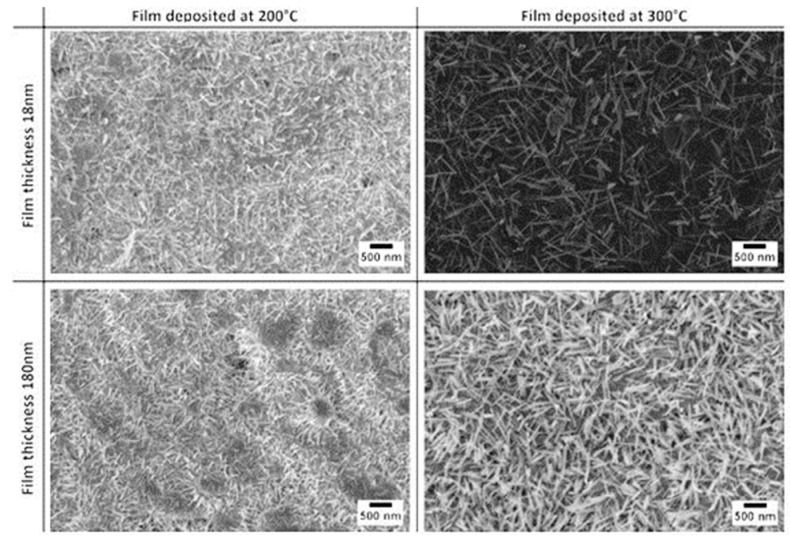
SEM pictures of oxidized 18 nm and 180 nm tungsten films deposited at different temperatures by RF magnetron sputtering. Reprinted from [22] with permission from The Royal Society of Chemistry.

Thickness and deposition temperature have a strong influence on nanowire growth. Figure 16 shows SEM images of WO_3_ with different morphologies and the average diameter of the WO_3_ is less than 40 nm for all the samples. The nanowires grown from the film deposited at 300 °C have bigger diameters and lengths compared to the others. Moreover, the average diameter increases by increasing the thickness of the films deposited at 300 °C. Concerning the layers deposited at 200 °C, the thickness has no impact on diameter, but it influences the nanowires’ density.

## 5. Chemical Sensing

One of the main activities carried out at the SENSOR Laboratory is the investigation of the ability of metal oxide nanostructures to detect gaseous species. In particular, the studies on quasi-1D nanostructures began in 2002 [73], when SnO_2_ nanobelts were proposed and integrated for the first time into conductometric sensors giving the scientific community a new and promising product for sensing applications. SENSOR is always focusing on the application of metal oxides materials and on their reliable integration into devices, therefore the efforts are focused on the direct growth on the active transducers to avoid difficult transfer processes from the growth to the transducers substrate, and moreover to improve the electrical and mechanical stability of the final device (a mandatory requirement for any eventual commercialization). Conductometric sensors are prepared depositing platinum interdigitated contacts by DC magnetron sputtering on the nanostructures and a platinum-heating element on the opposite side of the substrates. In this review, we report a comparison among the ability of the metal oxide 1D nanostructures prepared at SENSOR to detect the presence of gases.

### 5.1. Indium Oxide

In 2006, Bianchi et al. [29] studied the ability of nanowires and thick wire devices to detect different gaseous species, like NO_2_ and ethanol. The results showed the better performance of nanowire devices, which are able to detect all the tested species. NO_2_ and ethanol at high concentration are the only two gases detected by thick wire sensors. 

**Figure 17:**
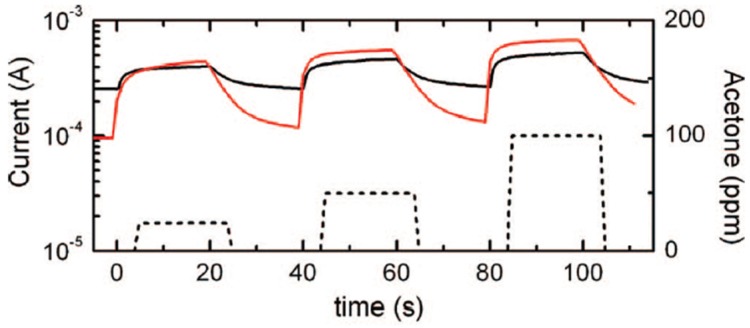
Kinetic variation of current of In_2_O_3_ nanowires (**red**) and thick wires (**black**) towards 25, 50, and 100 ppm of acetone (**dotted line**) at 40% relative humidity, 20 °C ambient temperature, and 400 °C operating temperature. Reprinted with permission from [34]. Copyright American Chemical Society.

The following year Vomiero at al. [34] performed gas-sensing analyses of NO_2_ and acetone on both thick wires and nanowires made of indium oxide. Concerning acetone, measurements were carried out at different gas concentrations (25, 50 and 100 ppm) resulting in a higher response in the case of nanowires devices. Figure 17 shows the dynamic current variation of nanowires and thick wires obtained at an operating temperature of 400 °C, which is in line with *n*-type semiconductor behaviour for both types of sensors.

The optimal working temperature for 25 ppm of acetone was found to be around 400 °C for both the morphologies. At this temperature, the response of nanowires is seven times higher than the thick wires’ one, because of the higher surface to volume ratio, emphasizing that the reduction of the wires lateral dimensions leads to an increase of the gas sensing capability. In the case of 500 ppb of NO_2_, the response of multiple thick wires device was not significant at temperatures higher than 300 °C, while nanowire gas sensors were able to detect nitrogen dioxide up to 400 °C. Also in the case of NO_2_, the chemical sensor based on nanowires performed better. 

The same year, another study by Vomiero et al. was carried out on In_2_O_3_ nanowires, grown starting from In seeds [28]. The analysis was carried out towards different concentrations of acetone (25, 50, 100 ppm) and the optimal working temperature was confirmed to be 400 °C. The sensor conductance variation (response) towards 25 ppm of acetone measured about 7 for this working temperature. The nanowire morphology shown in this work pointed out the reproducibility of the growth by the thermal evaporation technique. Gas sensing results reported in [34] and [28] are comparable, proving the capability of these nanostructures in the detection of acetone.

### 5.2. Tin Oxide

**Figure 18:**
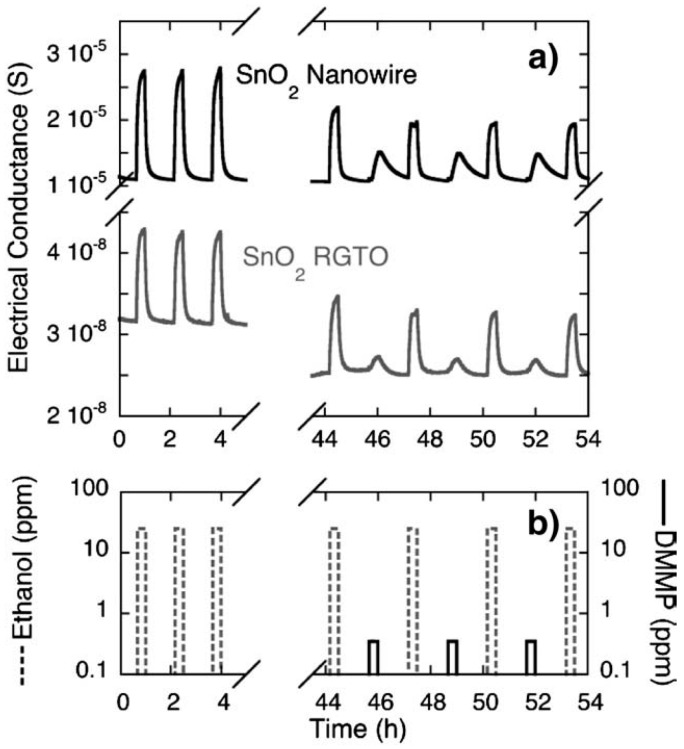
(**a**) Dynamic response of SnO_2_ nanowires (black line) and RGTO (grey line) sensors to (**b**) different injections of ethanol (25 ppm) and DMMP (0.2 ppm). The comparison between the ethanol sequence and the ethanol-DMMP sequence evidences the poisoning effects due to DMMP exposure, also at weak concentrations. Reprinted from [41] with permission from Elsevier, copyright (2009).

In 2008 a paper about the development of Pt/SnO_2_ nanowires/SiC MOS devices for hydrogen detection was published in collaboration with the Royal Melbourne Institute of Technology [37]. These devices were exposed to different concentrations of H_2_ (from 0.06% to 1%) with a working temperature ranging from 200 to 650 °C. The gas flow was set to 200 mL/min and a constant current of 1 μA was applied to the sensor during measurements. The response was calculated as voltage shift: at 0.25% of hydrogen is 70 mV at 420 °C, and 95 mV at 530 °C. Sensing performances increased with the operating temperature. Moreover, Sberveglieri et al. [41] proved in 2009 the ability of SnO_2_ nanowires to detect Chemical Warfare Agents (CWAs) like DMMP and acetonitrile, focusing the attention on DMMP and its poisoning effects. The authors compared the detection performance of SnO_2_ nanowires and thin films based gas sensors. According to the previous work [74], 500 °C was used as optimal operating temperature for tin oxide nanowires, and 400 °C for thin film. Nanowire sensors performed better than thin film ones. The high reactivity of DMMP molecules resulted in an appreciable response already at low concentrations (0.1 ppm) but, at the same time, it caused a drift in the electrical properties due to its poisoning effect. Both devices showed a decrease in the response towards ethanol after DMMP exposure (Figure 18). 

Recently, in 2014 Karakuscu et al. [43] showed the possibility to build “filter-sense foam”, a new device that can be used simultaneously as a filter and sensor. Gas sensing tests were carried out on SnO_2_ nanowires, SnO_2_ nanowires grown on SiC foam (SiC/SnO_2_) and SiC foam. Measurements were performed at room temperature (RT), in a dry and humid (RH = 30%) air atmosphere. The response of the sensors was investigated towards different concentration of NH_3_ (from 10 to 50 ppm) and NO_2_ (from 1 to 5 ppm). SiC foam was able to detect NH_3_ at RT, but towards NO_2_ it gave no response, the opposite behaviour was registered for SnO_2_. The new type of device showed behaviour comparable to the SiC foam one. An interesting result was achieved under UV light, because the filter-sense foam showed a combined SnO_2_ and SiC foam performance. The UV activation made this device able to detect NO_2_ as well, while it caused an increase in the conductance value and it improved the recovery time in the case of SnO_2_ and SiC foam. A humid air atmosphere enhanced the conductance of bare SiC foam and SiC/SnO_2_ in comparison with the conductance value under dry air. Bare SnO_2_ showed a comparable conductance value with and without humid air. 

### 5.3. Zinc Oxide

**Figure 19:**
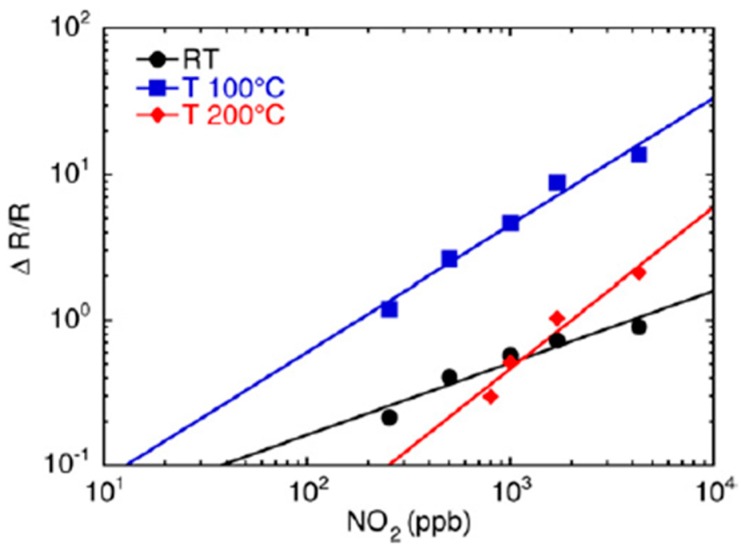
Response of zinc oxide nanowires as a function of the concentration of nitrogen dioxide introduced into the test chamber for RT and working temperatures of 100 and 200 °C. Reprinted from [45] with permission from IOP Publishing. All rights reserved.

In 2007, Comini et al. [45] proposed the integration of single crystal nanowires of zinc oxide into chemical sensors. Tests were performed towards different concentrations of nitrogen dioxide from RT to 200 °C. ZnO is a *n*-type semiconductor and it is confirmed by the dynamic response of the sensor towards NO_2_, showing a decrease of conductance in the presence of the gas. The recovery of the baseline value after the gas injections is complete and this is the result of a reversible reaction between the ZnO surface and gas molecules at this operating temperature. Figure 19 shows the calibration curves towards NO_2_ at RT, 100 and 200 °C. The trend of all the curves follows the metal oxide semiconductor sensor power law: S = A[Gas]^B^ [75] and it underlines that the gas detection ability increases by raising the temperature.

The same year Comini et al. [46] studied the gas sensing properties of zinc oxide nanowires grown by the thermal evaporation technique using zinc powder as source. The attention was focused on the sensing properties towards acetone (10, 25, 50 ppm) and ethanol (100, 250, 500 ppm). The reactions occurring between gas molecules and the sensing surface were reversible. These devices were able to detect acetone and ethanol at low concentrations (below 1 ppm) at 400 °C. The ability to recognize these two gases at low concentration is interesting for medical applications, like investigations for diabetes detection. Indeed, using the relationship between acetone and ethanol in exhaled breath and the glucose level in blood it is possible to identify this disease [76,77]. In the case of acetone, if the concentration in the exhaled breath is between 1.7 and 3.7 ppm it means that the person is diabetic [76]. Ethanol is a product of the alcoholic fermentation of glucose by gut bacteria. In healthy people the ethanol level is very low (0.02–0.04 mg/dL), while in people suffering from diabetes, the presence of ethanol is higher [77]. 

In 2012 a study on functionalised ZnO nanowire gas sensors was performed [50]. Samples were functionalized with two organic molecules: tris(hydroxymethyl)aminomethane (THMA) and dodecanethiol (DT). In particular, organic-coated ZnO nanowires and ZnO nanowires covered with a sparse layer of organic-coated ZnO nanoparticles were prepared, realizing two sensor configurations. Gas sensing tests were carried out exposing these devices to ammonia, nitrous oxide and nitrogen dioxide at an operating temperature of 190 °C. The results of the measurements showed that these samples were able to detect only NO_2_. In particular, the sensor behaviour remained unchanged after the functionalization with THMA, while the baseline value changed drastically after the organic coating. Tests carried out on DT-functionalized devices showed a consistent increase of the conductivity of all the sensors (above the full scale of instrument). In the case of ZnO nanowires covered with a sparse layer of organic-coated ZnO nanoparticles, the baseline exhibits a small change for both these organic molecules.

**Figure 20:**
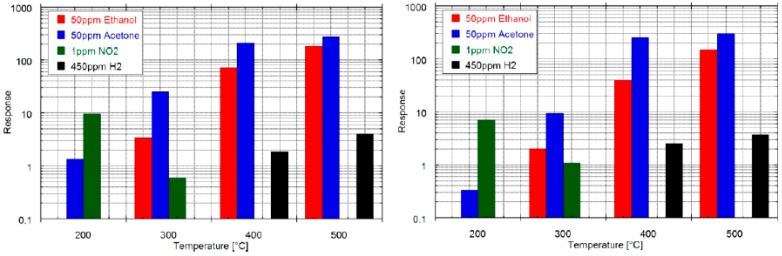
Sensitivity towards target gases at various temperatures for ZnO T400 (**left**) and ZnO T500 (**right**). Relative humidity was set at 50% at 20 °C. Reprinted from [16] with permission from IOP Publishing. All rights reserved.

At SENSOR, zinc oxide nanowires grown by thermal oxidation at two different oxidation temperatures (400 and 500 °C) [16] were tested at several concentrations of acetone, ethanol, hydrogen and nitrogen dioxide in the 200–500 °C temperature range. Measurements were performed maintaining the flow at 500 sccm and relative humidity at 50%. Gas sensing results highlighted that the responses of both device types towards all the chemical species are comparable (Figure 20). The optimal working temperature was 200 °C for NO_2_ and 500 °C for acetone, ethanol and hydrogen for both the gas sensor types. Moreover, the sensors’ reproducibility, i.e., the ability to produce comparable devices in terms of morphology and functional properties, was evaluated. Different batches of samples of ZnO were prepared and tested, showing a high reproducibility, which is an important starting point for possible commercialization. In the case of acetone, ethanol and hydrogen the evaluated detection limits considering 0.5 as minimum response are around 1 ppm, while for NO_2_ is around 200 ppb, which is higher than the European Environment Standards (99 ppb) [78]. H_2_ has a flammability range in air from 4% to 74%, for this reason a detection limit of 1 ppm is very attractive to ensure safety in various environments [79].

### 5.4. Nickel Oxide

**Figure 21:**
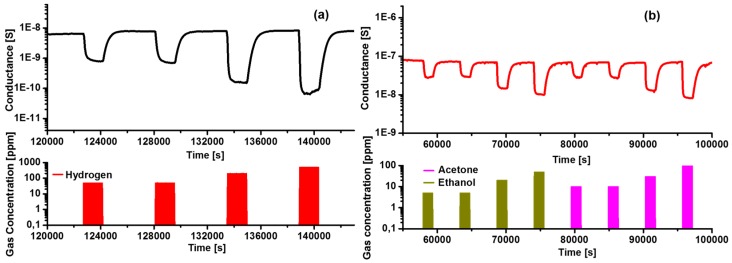
Dynamic response of NiO sensing device towards reducing gases. (**a**) (H_2_ red color; 50-50-200-500 ppm at 300 °C) and (**b**) (ethanol green color; 5-5-20-50 ppm at 500 °C), (acetone, pink color; 10-10-30-100 ppm at 500 °C) measured at relative humidity 50% at 20 °C. Reprinted from [52] with permission from IOP Publishing. All rights reserved.

In 2016 Kaur et al. [52] presented the integration of nickel oxide nanowires obtained by the VLS growth technique into gas sensing devices. The growth of NiO nanowires using Au as catalyst on alumina substrates was achieved. These nanowires were exposed to different concentrations of hydrogen, acetone, ethanol and carbon monoxide. Measurements were carried out at a constant flow of 200 sccm under 50% of humidity and a working temperature ranging from 200 °C to 500 °C. This temperature screening underlined the importance of different operating temperatures. Thus, acetone and ethanol were detected well at 500 °C while CO and H_2_ required 300 °C. Nickel oxide is a *p*-type material and Figure 21, which reports the dynamic responses of nanowires towards hydrogen, acetone and ethanol, confirms this point. In particular, the conductance value decreases when the semiconductor is exposed to reducing gases.

The influence of gas concentration on the response was studied for all the target gases at their corresponding optimal working temperature. The detection limit, evaluated considering the minimum response equal to 1, was 19 ppm for acetone, 10 ppm for ethanol and 6 ppm in case of hydrogen.

### 5.5. Copper Oxide

Zappa et al. [61] investigated in 2013 the preparation of copper oxide nanowires by thermal oxidation and their use as gas sensors. Measurements were carried out under a constant flow of 500 sccm and with a relative humidity of 50%. Interesting properties were found when CuO nanowire sensors were exposed to H_2_S, showing a dual behaviour towards different H_2_S concentrations. Adsorbed oxygen on the surface resulted in oxidation of H_2_S in the case of low concentrations of the chemical species, while the formation of CuS occurred covering the nanowire surface under high concentrations [80,81]. The first effect is dominant at high working temperatures while at lower temperatures (200–300 °C) CuS becomes dominant and it causes an increase of the electrical conductance. The gas sensing performance was investigated for other gases showing good capability for the detection of ozone, ethanol, hydrogen and acetone at 400 °C. The detection limits (considering 0.5 as minimum response) towards O_3_ and ethanol were 1 ppb, a lot below the ozone limit value indicated in European Standard, which is around 57 ppb [78], and lower than the presence of ethanol in healthy people breath (around 200 ppb) [77].

### 5.6. Tungsten Oxide

Tungsten oxide nanowires grown by thermal oxidation for chemical detection were studied in 2015 by Zappa et al. [22]. Measurements were carried out using a constant flow of 200 sccm with a relative humidity of 50%. Gas sensing tests were performed on samples prepared under different conditions, but the experiments showed similar behaviour, highlighting a slightly higher performance of the nanowires grown from 180 nm metal films. These samples were characterized by a higher nanowire density and an increased average diameter, in comparison to their counterparts. CO, NO_2_ and NH_3_ were well detected at temperatures below 300 °C, ethanol at 300 °C and acetone at 400 °C. The detection limit (considering 1 as the minimum response appreciable) of CO, NO_2_ and NH_3_ was 13 ppm, 1 ppm and 1.5 ppm respectively. The detection limit of CO is in line with the European Environment Standards that indicates a limit of 8 ppm c.a., while that of NO_2_ is higher (99 ppb) [78]. In the case of ammonia, the threshold limit value (toxicity) is 25 ppm [79] well above the detection capability of WO_3_ nanowires.

### 5.7. Results Comparison

Table 3 summarizes the different metal oxides investigated, the growth conditions of the nanostructures and the detected chemical species. One of the most studied chemical species is nitrogen dioxide and it was detected by all the tested conductometric sensors at low working temperatures (RT–200 °C). ZnO nanowires grown by the thermal oxidation technique [16] showed a lower detection limit than tungsten oxide nanowires (200 ppb versus 1 ppm) [22], but their operating temperature was slightly higher than that of WO_3_ ones (200 °C versus 100 °C). 

Acetone and ethanol were also investigated. These two gases were easily detected in a temperature range between 300 °C and 500 °C by all the tested materials. Zinc oxide nanowires grown by thermal evaporation techniques exhibited a detection limit below 1 ppm for acetone and ethanol at a working temperature of 400 °C [46]. 

These results are comparable with those of ZnO nanowires grown by a thermal oxidation process [16], which were characterized by a detection limit of 1 ppm for both chemical species, but at a higher operating temperature. Zinc oxide nanowires showed higher performance compared to NiO nanowires. In fact, gas sensors based on nickel oxide nanowires [52] exhibited a detection limit for acetone 19 times higher than the one of ZnO, and 10 times higher in the case of ethanol.

Measurements of hydrogen were performed on a Shottky type [37] sensor and on conductometric devices [16,52]. In the first case, the electrical characterization was performed on the Schottky contact formed between the metal oxide and the metal. The response was evaluated as a voltage shift, which depends on the decrease of Schottky barrier height, due to the electric field caused by the formation of an electrically polarized layer at the interface between metal-insulator and oxide. There is a direct relationship between temperature and voltage shift, in fact, when the first one increases (530 °C) the second one shows the same behaviour [37]. Concerning conductometric sensors, ZnO [16] and NiO [52] showed interesting results in the detection of hydrogen. The ability to recognize hydrogen was evaluated as a change in the electrical conductance value for both the materials. Zinc oxide nanowires prepared by thermal oxidation showed an optimal working temperature at 500 °C and a detection limit of 1 ppm, while for nickel oxide the optimal operating temperature was 300 °C and the detection limit was 6 ppm. In this case, the zinc oxide device is the best performing concerning the limit of detection, but it requires a higher operating temperature. 

The detection of carbon monoxide was evaluated for tungsten oxide nanowires [22] and nickel oxide nanowires [52]. In the first case, the optimal working temperature was found to be around 200 °C with a detection limit of 13 ppm, while for the other one the operating temperature was 300 °C. Ammonia was well detected by WO_3_ nanowire gas sensors [22], at working temperatures below 300 °C and with a detection limit of 1.5 ppm. The combination of SnO_2_ nanowires and a SiC foam substrate allowed the detection of this gas at room temperature, with and without UV activation [43]. 

Moreover, tin oxide nanowires showed interesting properties during measurements of CWAs, like DMMP. Low concentrations of this compound (0.1 ppm) were well detected by SnO_2_ nanowires at 500 °C [41]. 

On the other hand, copper oxide exhibited better performance towards other gases, like ozone, ethanol and hydrogen. The detection limits towards ozone and ethanol at 400 °C were 1 ppb and 10 ppm, respectively. At the same working temperature (400 °C) a CuO nanowire device gave a response below 1 for H_2_S@1 ppm, CO@500 ppm, NO_2_@0.5 ppm, and CH_4_@50 ppm [61].

All these materials showed interesting properties in the detection of different gases. ZnO nanowires prepared by the thermal evaporation technique exhibited the best results in the detection of NO_2_ [45,50]. Unfortunately, this limit is still slightly above the threshold set in the European standards. Concerning acetone and ethanol, zinc oxide nanowires showed the best results regardless of the preparation technique [16,46].

Gas sensors based on zinc oxide nanowires also exhibit interesting properties for the detection of hydrogen, showing a detection limit lower than the flammability range in air that makes them very attractive to ensure safety in different environments [16]. Other materials like NiO [52] and In_2_O_3_ [28,34] showed interesting properties towards these gases, but they performances are worss than those of ZnO.

In order to detect O_3_ in environmental samples CuO nanowire-based gas sensors showed interesting properties, with a detection limit lower than the threshold of the European standards [61].

WO_3_ nanowire-based gas sensors can be interesting tools to control the level of ammonia and carbon monoxide in the environment. This is possible thanks to detection limits in line with the European standards [22].

In conclusion, the best performing gas sensors are based on ZnO nanowires concerning the detection of NO_2_, acetone and ethanol, while the other materials are important to ensure a good control for the presence of other gaseous species (NH_3_, CO, O_3_, etc.) in the environment.

## 6. Conclusions

We have summarized the multiannual research performed at the SENSOR Lab (Brescia) to develop low cost and power, simple and reliable devices based on metal oxides, one among the “most promising” technologies for chemical sensing, thanks to simplicity of the transduced physical quantity and the possibility of integration into Si technology. Since early in the century we have made strong efforts to prepare and characterize a wide spectrum of oxides by simply evaporating commercial MOX powders at high temperatures. High degrees of crystallinity and atomically sharp terminations make nanowires very suitable candidates for the development of a new generation of stable chemical sensors. For each material and structure, we have collected and determined the performance for a wide spectrum of chemical sensing applications.

## Figures and Tables

**Table 1:** Deposition conditions of metallic thin films.

Material	Power	Temperature	Film Thickness
CuO	50 W	200, 300, 400 °C	300 nm–3 µm
ZnO	50 W	Room Temperature	750 nm–4.5 µm
WO_3_	100 W	200, 300 °C	18 nm–180 nm

**Table 2:** Oxidation conditions for metallic layers.

Material	Time	Pressure	Temperature	Gas Flow
CuO	15 h	Atmospheric pressure	300–400 °C	300SCCM (80% O_2_ and 20% Ar)
ZnO	12 h	Atmospheric pressure	200–900 °C	300SCCM (20% O_2_ and 80% Ar)
WO_3_	1 h	0.8 mbar	550 °C	O_2_-2SCCM

**Table 3:** Summary on the different materials investigated, the growth conditions of the prepared nanostructures and the detected chemical species.

Metal Oxide	Growth Technique		Growth Conditions					Chemical Sensing	Ref.
			Source	E_t_ (°C)	S_t_ (°C)	P (mbar)	Growth Catalyst		
In_2_O_3_	VS	nanowires	In_2_O_3_ powder	1500	800–1100	200		Ethanol (30 ppm@200 °C): ~0.97 (thick wires); NO_2_ (5 ppm@200 °C): ~6.21 (nanowires), ~0.86 (thick wires)	[29]
		thick wires							
	VS	nanowires	In_2_O_3_ powder	1500	750–800	200	In	NO_2_ (500 ppb@300 °C): ~0.4 (nanowires), ~0.1 (thick wires); acetone (25 ppm@400 °C): ~3.7 (nanowires), ~0.5 (thick wires)	[34]
		thick wires							
	VS		In_2_O_3_ powder	1500	700–1000	200	In	Acetone (25 ppm@400 °C): ~7	[28]
SnO_2_	VLS		SnO_2_ powder	1370	400–500	100	Pt	Hydrogen (0.25%): 70 mV@420 °C, 95 mV@530 °C	[37]
	VLS		SnO_2_ powder	1370	430–470	100	Pt	DMMP (0.2 ppm): ~0.3 (NWs@500 °C), ~0.04 (thin film@400 °C); acetonitrile (1 ppm): ~0.2 (NWs@500 °C), 0.04 (thin film@400 °C); ethanol (25 ppm): ~1.5 (NWs@500 °C), 0.4 (thin film@400 °C)	[41]
	VLS		SnO_2_ powder	1370	500	100	Au	NO_2_ (5 ppm@RT): ~0.2; NH_3_ (50 ppm@RT): ~0.35	[43]
ZnO	VS		ZnO powder	1370	400–500	100		NO_2_ (500 ppb@100 °C): ~3	[45]
	VS		Zn powder	600	3–5 mm from the powder	Ambient pressure (Ar and O_2_ 21%)		Acetone (50 ppm@400 °C): ~35 ethanol (500 ppm@400 °C): ~65	[46]
	VS *		Zn powder	600	3–5 mm from the powder	hundreds		NH_3_, N_2_O (no significant response); NO_2_ (2 ppm@190 °C): ~0.5 (THMA), ~0.2 (pure)	[50]
	Thermal Oxidation		Zn layer (4.5 μm)	400, 500	400, 500	100% O_2_		Acetone (50 ppm@500 °C): ~300 (ox@400 °C, ox@500 °C); ethanol (50 ppm@500 °C): ~200 (ox@400°C), ~150 (ox@500 °C); H_2_ (450 ppm@500 °C): ~4 (ox@400 °C, ox@500 °C); NO_2_ (1 ppm@200 °C): ~10 (ox@400 °C); 7 (ox@500 °C)	[16]
NiO	VLS		NiO powder	1400	900–1000	1	Au	Acetone (30 ppm@500 °C): ~1.3; ethanol (20 ppm@500 °C): ~1.5; H_2_ (50 ppm@300 °C): ~8; CO (20 ppm@300 °C): ~0.3	[52]
CuO	Thermal Oxidation		Cu layer (1.8 μm)	300	300	80% O_2_ 20% Ar		Acetone (100 ppm@400 °C): >1; ethanol (500 ppm@400 °C): >1.5; H_2_ (5000 ppm@400 °C): >1; H_2_S (1 ppm@400 °C): <0.5; O_3_ (300 ppb@400 °C): ~2.5; CH_4_ (50 ppm@400 °C): <0.5; CO (500 ppm@400 °C): >0.5	[61]
WO_3_	Thermal Oxidation		W layer (18 nm, 180 nm)	550	550	0.8		Acetone (100 ppm@400 °C): ~10; ethanol (50 ppm@300 °C): ~5; NO_2_ (9 ppm@300 °C): ~20; CO (500 ppm@200 °C): ~70; NH_3_ (5 ppm@200 °C): ~10	[22]

* Functionalised by ZnO, THMA-ZnO and DT-ZnO.
